# Picture free recall performance linked to the brain's structural connectome

**DOI:** 10.1002/brb3.721

**Published:** 2017-05-23

**Authors:** David Coynel, Leo Gschwind, Matthias Fastenrath, Virginie Freytag, Annette Milnik, Klara Spalek, Andreas Papassotiropoulos, Dominique J.‐F. de Quervain

**Affiliations:** ^1^ Division of Cognitive Neuroscience Department of Psychology University of Basel Basel Switzerland; ^2^ Transfaculty Research Platform University of Basel Basel Switzerland; ^3^ Division of Molecular Neuroscience Department of Psychology University of Basel Basel Switzerland; ^4^ Psychiatric University Clinics University of Basel Basel Switzerland; ^5^ Department Biozentrum Life Sciences Training Facility University of Basel Basel Switzerland

**Keywords:** centrality, connectome, diffusion MRI, interindividual differences, memory, volumetry

## Abstract

**Introduction:**

Memory functions are highly variable between healthy humans. The neural correlates of this variability remain largely unknown.

**Methods:**

Here, we investigated how differences in free recall performance are associated with DTI‐based properties of the brain's structural connectome and with grey matter volumes in 664 healthy young individuals tested in the same MR scanner.

**Results:**

Global structural connectivity, but not overall or regional grey matter volumes, positively correlated with recall performance. Moreover, a set of 22 inter‐regional connections, including some with no previously reported relation to human memory, such as the connection between the temporal pole and the nucleus accumbens, explained 7.8% of phenotypic variance.

**Conclusions:**

In conclusion, this large‐scale study indicates that individual memory performance is associated with the level of structural brain connectivity.

## 1 INTRODUCTION

Much about the group‐level neuroanatomical basis of episodic memory functions is known today from studies in patients with brain lesions and from imaging studies investigating brain activations related to memory processes in healthy humans (Battaglia, Benchenane, Sirota, Pennartz, & Wiener, 2011; Cabeza, Ciaramelli, Olson, & Moscovitch, 2008; Eichenbaum, 2000; Kragel & Polyn, 2015; Rugg & Vilberg, 2013; Uncapher, Hutchinson, & Wagner, 2011; Watrous, Tandon, Conner, Pieters, & Ekstrom, 2013). However, large interindividual differences in memory performance are observed, even across healthy individuals (de Quervain et al., 2003). Little is known about the neuroanatomical basis of such behavioral variability. It might be explained, at least partly, by individual differences in white‐matter properties, given their link to memory disorders (Metzler‐Baddeley et al., 2012; Pievani, Filippini, van den Heuvel, Cappa, & Frisoni, 2014). In this context, previous studies related diffusion characteristics such as fractional anisotropy (FA) to individual differences in episodic memory performance, both for well‐known white matter pathways (Rudebeck et al., 2009) or at the voxel‐wise level (Fuentemilla et al., 2009). However, that approach might not be able to characterize how inter‐regional connections relate to behavior. For this purpose, brain connectomics has proven to be a relevant approach to studying brain connectivity (Behrens & Sporns, 2012), both from functional and structural perspectives. The brain is modeled as network, or a graph, where each brain region is represented by a node, and the edges of the graph represent inter‐regional connections. These edges can represent both functional or structural connections. Complex behavior is associated with a dynamic repertoire of functional interactions, so far mainly studied during rest (Sporns, 2014), which are related to structural connections. The structural connectome can be reliably investigated in vivo by using diffusion imaging‐based tractography (Bassett, Brown, Deshpande, Carlson, & Grafton, 2011; Owen et al., 2013). The structural connectome may provide a basis to explain interindividual differences in behavior (Behrens & Sporns, 2012; Johansen‐Berg, 2010, 2012). Interindividual variability in the structural connectome has been associated with intellectual performance in healthy young adults (Li et al., 2009) and in aging individuals (Fischer, Wolf, Scheurich, & Fellgiebel, 2014). But it is not yet known whether or not specific neurocognitive systems, such as episodic or working memory, exhibit similar patterns. Furthermore, as low sample sizes can be detrimental in obtaining reliable effect size estimations (Button et al., 2013), large cohorts are needed in order to better understand interindividual variability in complex behavior.

In the present study of a large cohort of 664 subjects, we focused on relations between the structural connectome and free recall performance of previously seen IAPS pictures (Lang, Bradley, & Cuthbert, 2008). Even though several mechanisms might be involved in successful free recall (Dickerson & Eichenbaum, 2010), this internally cued retrieval process is thought to depend mainly on recollection (Squire & Wixted, 2011). Consequently, free recall allows us to assess one aspect of the multi‐dimensional processes that underlie episodic memory. This test can be complemented by tests of cued recall or recognition, which inform us about item familiarity. The IAPS normalized picture system has been used previously to characterize free recall (Heck et al., 2015) as well as emotional memory (Dolcos, LaBar, & Cabeza, 2004; Hofstetter, Achaibou, & Vuilleumier, 2012). Emotional material is usually better remembered than neutral material (McGaugh, 2000), and functional interactions during encoding and retrieval have been shown to be affected by emotional valence (Hermans et al., 2014; Kark & Kensinger, 2015). However, it is not known whether or not structural network properties are related to individual variability in emotional memory. The stimuli used in this study, containing both emotional and neutral pictures, consequently allowed us to test the potential impact of emotional valence on brain‐behavior relationships. An additional working memory task (N‐Back) served as a non‐episodic control task.

Several aspects of brain connectivity were assessed. We first focused on characterizing inter‐regional connections: the average connection strength of a node to the rest of the network is called *degree*, which can in turn be generalized to a metric called *network cost*, which is a simple estimator of physical wiring cost. We took advantage of this natural hierarchical representation of inter‐regional connections by investigating their association with memory performance at the whole‐brain level, at the regional level, and at the region‐to‐region level. Each higher‐resolution level was investigated only if the lower‐level null hypothesis was rejected (Duarte‐Carvajalino et al., 2012). While this approach avoids testing first at high resolutions, where the number of hypotheses to be tested can be very large, it can also hide certain effects that are too specific. As a consequence, this approach was complemented by the direct investigation of the association between memory performance and region‐to‐region connections in the network‐based statistic framework (Zalesky, Fornito, & Bullmore, 2010), which aims to identify connected components of a graph while controlling for family wise error rate. In this context, and given prior findings relating intelligence to the total number of edges (Li et al., 2009), we hypothesized that cost‐related metrics would be associated to free recall performance.

We also assessed other network properties, such as measures of segregation (*e.g*. clustering coefficient), centrality (*e.g*. betweenness centrality), or integration (*e.g*. global efficiency) (Rubinov & Sporns, 2010). The latter has been positively linked, for example, to individual differences in intelligence tests performance (Li et al., 2009). Again, those network characteristics were extracted at the global (network), regional, or region‐to‐region level (Duarte‐Carvajalino et al., 2012). In line with previous findings, we hypothesized that better free recall performance would be reflected by more effective networks properties (*e.g*. higher centrality and integration measures).

## 2 MATERIAL AND METHODS

### 2.1 Participants

A total of *N* = 679 participants of an ongoing study were included (275 males, 404 females; 22.85 ± 3.37 years old; dataset status April 2013). This large‐scale and ongoing study serves to address several scientific questions, including questions in the field of imaging genetics, where several papers on the dataset have been previously published (Harrisberger et al., 2014; Heck et al., 2014). The subjects were free of any lifetime neurological or psychiatric illness, and did not take any medication at the time of the experiment (except hormonal contraceptives). All subjects gave written informed consent before participation in the study. The ethics committee of the Canton of Basel, Switzerland, approved the study protocol. Complete datasets (behavior and structural imaging) for analysis were available from *N* = 664 participants (see below).

### 2.2 Episodic memory task

We used a picture free recall task to assess episodic memory. For picture encoding, 72 pictures, divided into three valence groups (negative, neutral, and positive), as well as 24 scrambled pictures were presented during the MRI scans by using MR‐compatible LCD goggles (VisualSystem, NordicNeuroLab). On the basis of normative valence scores, pictures from the International Affective Picture System (IAPS; [Lang et al., 2008]) were assigned to emotionally negative (2.3 ± 0.6), neutral (5.0 ± 0.3), and positive (7.6 ± 0.4) groups. Eight neutral pictures were selected from an in‐house standardized picture set in order to equate the picture set for visual complexity and content (*e.g*. human presence). Examples of pictures are as follows: erotica, sports, and appealing animals for the positive valence; bodily injury, snake, and attack scenes for the negative valence; and finally, neutral faces, household objects, and buildings for the neutral condition.

Pictures were presented in an event‐related design, for 2.5 s in a quasi‐randomized order so that a maximum of four pictures of the same category were shown consecutively. A fixation‐cross appeared on the screen for 500 ms before each picture presentation. Trials were separated by a variable inter‐trial period (period between appearance of a picture and the next fixation cross) of 9–12 s (jitter). During the inter‐trial period, subjects rated the presented pictures according to valence (negative – neutral – positive) and arousal (low – middle – high) on a three‐point scale. Four additional pictures showing neutral objects were used to control for primacy and recency effects in memory. The scrambled pictures consisted of a geometrical object in the foreground while the background contained the color information of all pictures used in the experiment (except primacy and recency pictures), overlayed with a crystal and distortion filter (Adobe Photoshop CS3). The object had to be rated regarding its form (vertical, symmetric, horizontal) and size (small, medium, large).

In an unannounced recall task outside of the scanner, subjects had to freely recall the previously presented pictures, 10 min after the end of picture encoding. An unannounced free recall test was used to avoid recall performance to be influenced by interindividual differences in learning strategies, potentially reflecting non‐mnemonic processes. Participants had to write down a short description (a few words) of the previously seen pictures. Primacy and recency pictures that were remembered as well as training pictures were excluded from the analysis. No time limit was set for this task. Two trained investigators independently rated the descriptions for recall success (interrater reliability >98%). No details were required for correct scoring as pictures were all distinct from each other. The total number of freely recalled pictures was defined as the episodic memory performance phenotype.

### 2.3 Working memory task

Subjects completed the 0‐ and 2‐back version of the n‐back task after picture encoding, and before the recall task (Heck et al., 2014). The task consists of 12 blocks (six 0‐back, six 2‐back), in which 14 test stimuli (letters) were presented. The 0‐back condition required participants to respond to the occurrence of the letter ‘x’ in a sequence of letters (*e.g*., N – l – X – g) and served as a non‐memory‐guided control condition, measuring general attention, concentration, and reaction time. The 2‐back condition required subjects to compare the currently presented letter with the penultimate letter to decide whether they are identical or not (*e.g*., S – f – s – g). This task requires online monitoring, updating, and manipulating remembered information. It is therefore assumed to involve key working memory‐related processes. Performance was recorded as a number of correct responses (accuracy). Performance in the 0‐back condition (mean accuracy) served as the phenotype reflecting attentional processes, and the difference in accuracy between the 2‐back and the 0‐back condition served as the phenotype reflecting working memory performance.

### 2.4 MRI acquisition

Measurements were performed on a Siemens Magnetom Verio 3T whole‐body MR unit equipped with a twelve‐channel head coil. A high‐resolution T1‐weighted anatomical image was acquired with a magnetization prepared gradient echo sequence (MPRAGE, TR = 2000 ms; TE = 3.37 ms; TI = 1,000 ms; flip angle=8; 176 sagittal slices; FOV = 256 mm; voxel size 1 × 1 × 1 mm^3^). Diffusion volumes were acquired by using a single‐shot echo‐planar sequence, and consisted of 64 diffusion‐weighted volumes (b = 900 s/mm^2^) and one unweighted volume (b = 0). Acquisition parameters were as follows: TR = 9,000 ms, TE = 82 ms, FOV = 320 mm, GRAPPA R = 2.0, voxel size 2.5 × 2.5 × 2.5 mm^3^.

#### 2.4.1 Anatomical T1‐weighted imaging analyses

After visual inspection, T1‐weighted images of fifteen participants were excluded due to excessive movement or scanner noise. Complete datasets (behavior and structural imaging) were available from *N* = 664 participants.

##### Preprocessing

T1‐weighted images were segmented into cortical and subcortical structures by using FreeSurfer v4.5 (Fischl et al., 2002) (RRID:SCR_001847). Labeling cortical gyri was based on the Desikan‐Killiany atlas (Desikan et al., 2006), yielding 34 regions per hemisphere. Eighty‐two regions (68 cortical and 14 subcortical) were subsequently considered as nodes for the network analyses. The binary masks defining these nodes were coregistered to the reference unweighted diffusion volume (b = 0) using FreeSurfer's *bbregister* command, initialized with the *spm_coreg* command from SPM8 (RRID:SCR_007037).

##### Statistical analyses

The association between memory performance and grey matter volumes was determined by Spearman correlation analyses. Memory performance was corrected for age and gender. This was achieved by considering as a new memory performance variable the residuals of a linear model including memory as a dependent variable and age/gender as independent variables. Similarly, volumes were corrected for age, gender and intracranial volume (as estimated by FreeSurfer's total intracranial volume).

#### 2.4.2 Diffusion‐weighted imaging analyses

**Figure 1:**
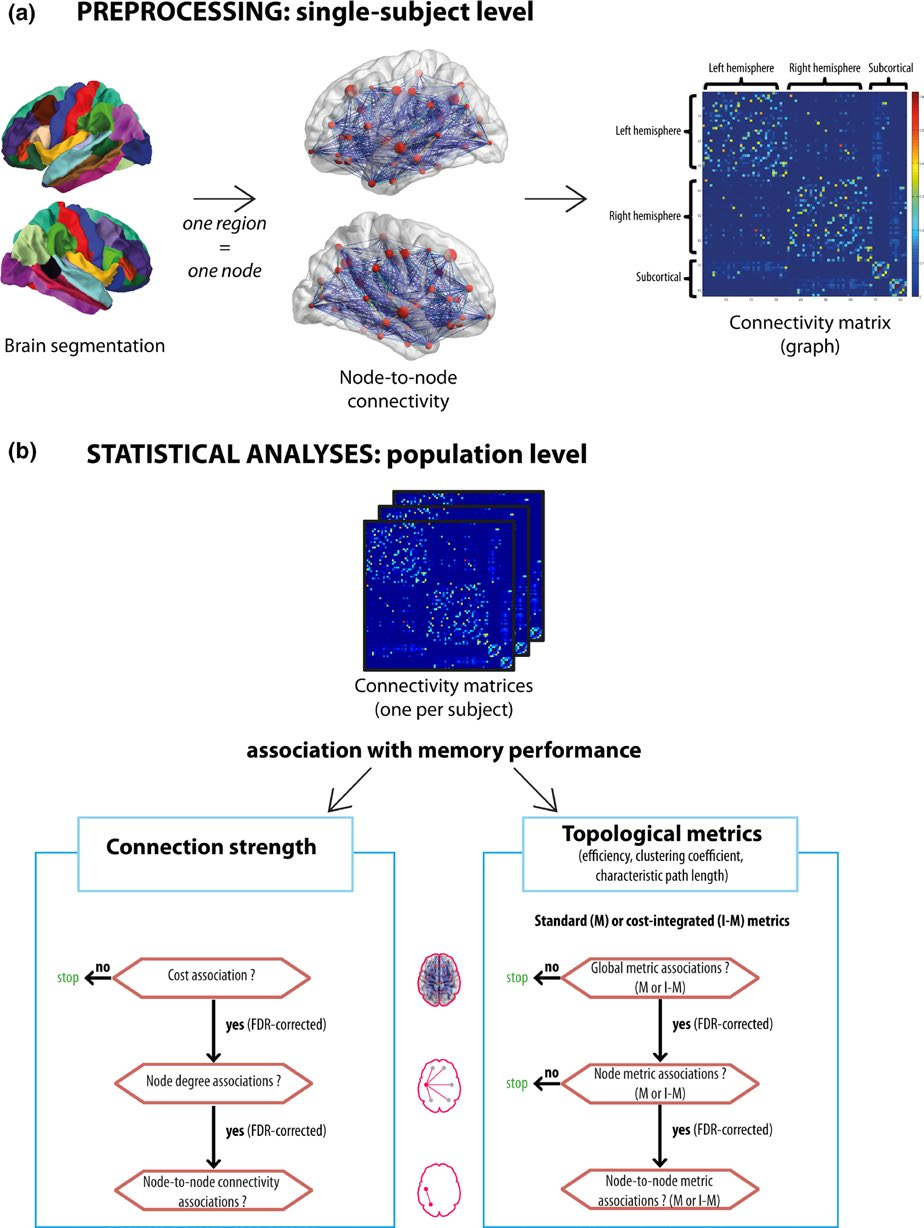
Analysis pipeline. (a) Summary of the preprocessing steps at the single‐subject level. Individual brain segmentation was used as the basis to compute a connectivity matrix from which global, regional, and node‐to‐node network characteristics were computed. (b) Summary of statistical analyses at the population‐level. Interindividual differences in network characteristics were related to memory performance. Association to network cost was tested first, followed by other topological metrics. Cost‐integrated metrics were computed if a relation to network cost was found. All tests were done following a hierarchical scheme, from global, to regional, to node‐to‐node level, using an FDR procedure at each level

The processing steps involved in this section are summarized in Figure 1a.

##### Preprocessing

Diffusion‐weighted images were pre‐processed by using FSL v4.1.7 (Jenkinson, Beckmann, Behrens, Woolrich, & Smith, 2012) (RRID:SCR_002823). Data of 76 participants, for whom slice corruption due to movement was detected (at maximum 2 directions), were corrected by removing the corrupted directions before further processing (Sharman et al., 2011). Images were first coregistered to the reference unweighted volume (b = 0) by using an affine transformation for the correction of head motion and eddy current induced image distortion. Voxelwise model fitting of diffusion orientations was then performed. The local probability distribution of fiber direction was estimated by using *bedpostx*, allowing for automatic estimation of multiple fiber directions within each voxel (at most two). This approach leads to better sensitivity in the detection of fiber populations as compared to single‐fiber or deterministic approaches (Behrens, Berg, Jbabdi, Rushworth, & Woolrich, 2007).

##### Structural brain network construction: single subject level, weighted connectivity matrix

Each FreeSurfer‐segmented region was considered as a node (see Figure 1a). Connectivity probability between nodes was estimated by using probabilistic tractography as implemented in *probtrackx2* in FSL v5.0.2 (Behrens et al., 2007). Each node was selected as a seed region, and five thousand sample streamlines were drawn from each voxel within the seed nodes. Each streamline followed local orientations sampled from the posterior distribution given by *bedpostx*. The streamline stopped when it reached another node, or was excluded when it left the brain or passed through the ventricles. The node‐to‐node connection probability was represented in a weighted fashion, computed as the number of streamlines successfully reaching another node, divided by the total number of drawn streamlines that were not excluded (Behrens et al., 2007). We focused our subsequent analyses on weighted networks to avoid a potential loss of information when studying binary networks, in which case all non‐null weights would have been set to 1. A whole‐brain symmetrical connectivity matrix was constructed for each subject by averaging the connectivity probabilities obtained from node *i* to *j* and from node *j* to *i* (Gong et al., 2009). In summary, we computed one 82 × 82 weighted connectivity matrix per subject that was used for subsequent analyses.

##### Structural brain network construction: population level, discarding spurious connections

A common connectivity threshold at the population level was used to discard spurious connections. More precisely, those connections *C*_*ij*_ for which, across subjects, mean(*C*_*ij*_)+2std(*C*_*ij*_) was below a connectivity value of 0.01 were excluded in all subjects (Gong et al., 2009).

#### 2.4.3 Diffusion‐weighted brain network characteristics

For each subject, brain network characteristics were computed with the Brain Connectivity Toolbox (Bassett et al., 2011; Rubinov & Sporns, 2010) (RRID:SCR_004841). G refers to a weighted connectivity matrix, *i.e*. a weighted graph, where R = 82 is the number of nodes in the graph and connections between nodes are referred to as *edges*. The analyses steps involved in this section are summarized in Figure 1b.

##### Network cost

The left side of Figure 1b represents the hierarchical approach relating connection strength to network cost. The network cost (or weighted network density) is defined as the sum of connection weights in G, normalized by the total possible number of edges (R*(R−1)/2) (Latora & Marchiori, 2003). This measure is a generalization of the nodal degree (or nodal strength), which is defined as the average connectivity of a node, across its R−1 connections. It represents a simple estimator of physical wiring cost.

##### Topological metrics: definitions

The right side of Figure 1b represents the hierarchical approach for the other network properties. A set of topological properties (Bullmore & Sporns, 2009) were computed in order to further quantify the structural networks. We furthermore indicate when measures can be generalized at different spatial scale (whole‐brain, regional, or node‐to‐node):

- Clustering coefficient: the fraction of a node's neighbors that are also neighbors of each other. This measure can be generalized to the whole‐brain level.
- Characteristic path length: the average shortest path length between all pairs of nodes. The value was normalized by the average characteristic path length of 100 comparable random networks (preserving the degree distribution, with approximately 20 rewirings per edge).
- Global efficiency: the average inverse shortest path length. It can be decomposed as the average nodal efficiency, computed on local subgraphs comprising neighbors of each node.
- Betweenness centrality: the fraction of shortest paths between any pair of edges that travel through the node. This measure can be generalized to the whole‐brain level.

##### Topological metrics: cost‐integrated metrics

It has been shown that differences in topology due to differences in cost, or cost‐dependency, can confound the comparison of different brain networks (Ginestet, Nichols, Bullmore, & Simmons, 2011). A proposed solution is to study cost‐integrated network characteristics, integrating over the whole range of possible costs. We therefore computed the cost‐integrated versions of the above‐mentioned topological metrics if a significant association was found between network cost and memory performance (Figure 1b, labeled I‐M metrics). Not controlling for differences in network cost might result in spurious associations with memory performance (*e.g*. with the clustering coefficient), and lead to a potentially misleading interpretation of the findings. One drawback of cost‐integrated metrics, on the other hand, is that they might fail to capture subtle interindividual differences that might occur only in a limited density range. Appropriate network comparison is still a topic of ongoing discussions (Fornito, Zalesky, & Breakspear, 2013; Ginestet et al., 2011; van Wijk, Stam, & Daffertshofer, 2010). As anatomical networks are sparse (*i.e*. not fully connected), we did not investigate the whole possible range of cost values (in theory between 1/(R*(R−1)/2) and 1), but values between 1/(R*(R−1)/2) and the smallest common value across all subjects for the maximum cost (0.3538). If no association were found between network cost and memory performance, we would have computed the standard topological metrics (Figure 1b, labeled M metrics).

##### Statistical analyses

The association between interindividual differences in memory performance and brain network properties was assessed by using linear mixed‐effects models and Spearman correlation. Spearman correlation is a non‐parametric measure of association better suited than Pearson correlation for brain‐behavior correlation analyses as it is less sensitive to outliers (Rousselet & Pernet, 2012). We also report 95% percentile bootstrap confidence intervals (CIs) (Wilcox, 2012), which are not often reported in such associations and might give important information about the reliability of the estimates. We first regressed out the effects of age and gender for memory performance and network properties, and additionally the effect of intracranial volume for the network properties. We did so by considering the residuals of a linear model including them as covariates.

Linear mixed‐effect models, as implemented in the R package nlme (Pinheiro, Bates, DebRoy, & Sarkar, 2012; R Core Team, 2012), were used to test for possible interaction effects between valence (positive, negative, and neutral pictures) and network properties on memory performance. Subjects were entered as random effect. If no significant interaction was present, a Spearman correlation coefficient was used to estimate the association between the global memory performance (positive+negative+neutral pictures) and the network property. Possible interaction effects between gender or age and global network properties on memory performance were assessed with linear models. Finally, to assess the combined effect of multiple connections on episodic memory performance, robust linear regression models with bisquare weight function, as implemented in the Matlab function *LinearModel.fit*, were employed.

We adopted a hierarchical approach in hypothesis testing, similar to the one proposed by (Duarte‐Carvajalino et al., 2012). Briefly, the brain network characteristics can be arranged in a hierarchical fashion from global (one measure per subject, lowest level in the hierarchy), to the node level (82 measures per subject), and to the node‐to‐node level (at most R*(R−1)/2 measures per subject, highest level in the hierarchy). In this framework, higher resolution hypotheses are tested with an FDR procedure at each level to control for multiple hypothesis testing, but only if the lower‐level null hypothesis is rejected (Duarte‐Carvajalino et al., 2012; Yekutieli, 2008). This avoids testing first at high resolutions, where the number of hypotheses to be tested can be very large. This procedure has the advantage of allowing us to identify global effects because of the reduced number of multiple comparisons. However, very specific effects may remain undetected if they do not impact the global measures sufficiently. In the case of the cost analysis, the nodal degree and the node‐to‐node connectivity values were the highest level of the hierarchy. For the topological metrics analysis, node‐specific characteristics, such as clustering coefficient or betweenness centrality, were averaged to create a global value that represented the lower level in the hierarchy.

##### Network‐based statistics

The hierarchical approach on the association between node‐to‐node connections and memory performance was complemented by a mass‐univariate approach as implemented in the Network‐Based Statistics toolbox (NBS) (Zalesky et al., 2010). Starting from the individual connectivity matrices, this approach aims to identify sets of connected regions while controlling for family wise error (FWE) rate. It consists of four main steps: (1) a test statistic is computed for each link, in this case a *t*‐test representing the association between the link and memory performance, including age, gender, and intracranial volume as covariates; (2) a threshold is selected to construct a set of suprathreshold links, and we employed a stringent threshold of *p *<* *.0063, corresponding to *T *=* *2.5; (3) connected components are identified by using a breadth first search algorithm, and the number of links it comprises is stored; (4) a permutation‐based *p*‐value is assigned to each identified component by indexing its size with the null distribution of maximal component size. Ten thousand permutations were computed, and the resulting significant components were identified at a cluster‐level FWE‐corrected *p*‐value of *p *<* *.005.

#### 2.4.4 Data visualization

Results were visualized with the PySurfer software (https://pysurfer.github.io, RRID:SCR_002524) and the BrainNet Viewer (Xia, Wang, & He, 2013) (http://www.nitrc.org/projects/bnv/, RRID:SCR_009446).

## 3 RESULTS

### 3.1 Global network characteristic

**Figure 2:**
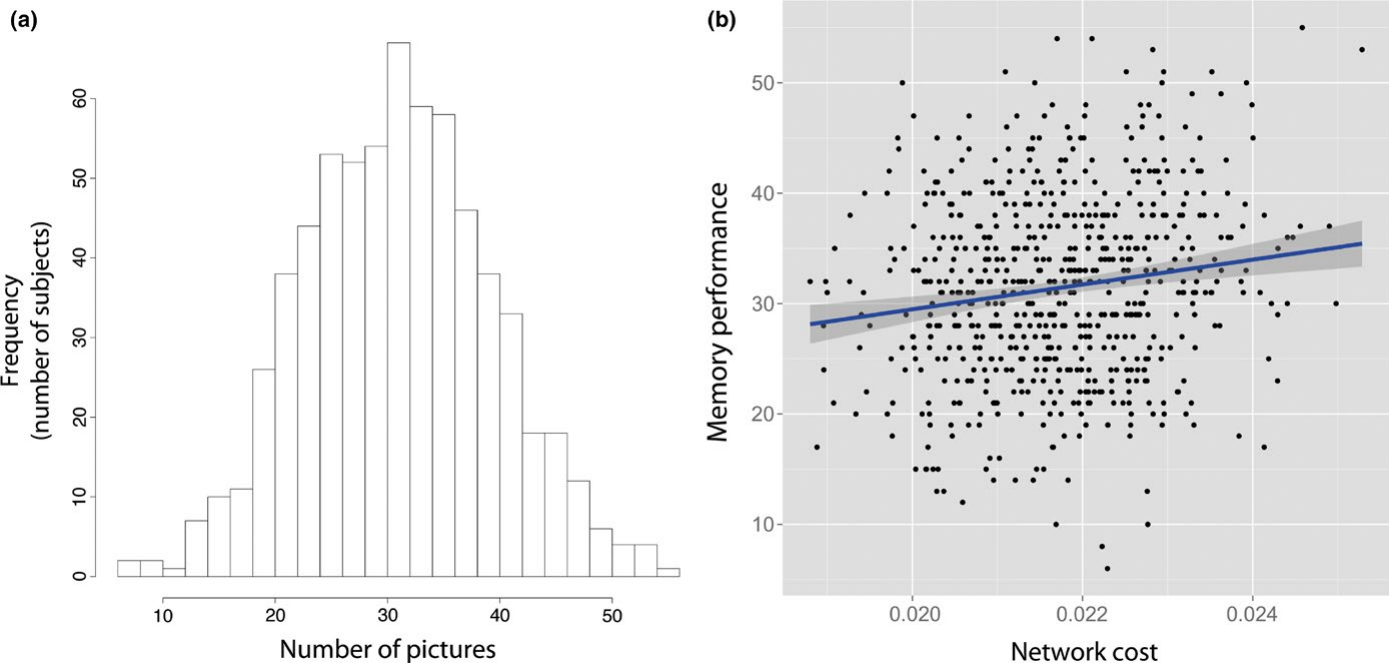
Picture recall performance. (a) Distribution of picture recall performance; (b) Association between network cost and picture recall performance

Free recall performance was positively correlated with network cost (Figure 2; Spearman *r *=* *.102; *p*_*nominal*_=.0086; *p*_*FDR*_=.043; coefficient of determination *R* ^*2*^=.011; 95% confidence interval [0.02,0.18]). This is a measure of global structural connectivity. There were no significant interactions with gender, age, or emotional valence of the stimulus material on this association (all *p *≥* *.39). Furthermore, we assessed whether or not other cognitive functions, such as attention or working memory, influenced this association. The relationship remained significant after controlling for attention (*r *=* *.098; *p *=* *.0117) or working memory performance (*r *=* *.097; *p *=* *.0125), indicating that the reported association does not depend on the assessed non‐episodic cognitive domains. These measures were themselves not significantly associated with network cost (*r*_*attention*_=.015; *p *=* *.7 and *r*_*working memory*_=.029; *p *=* *.46).

### 3.2 Region‐specific level

**Figure 3:**
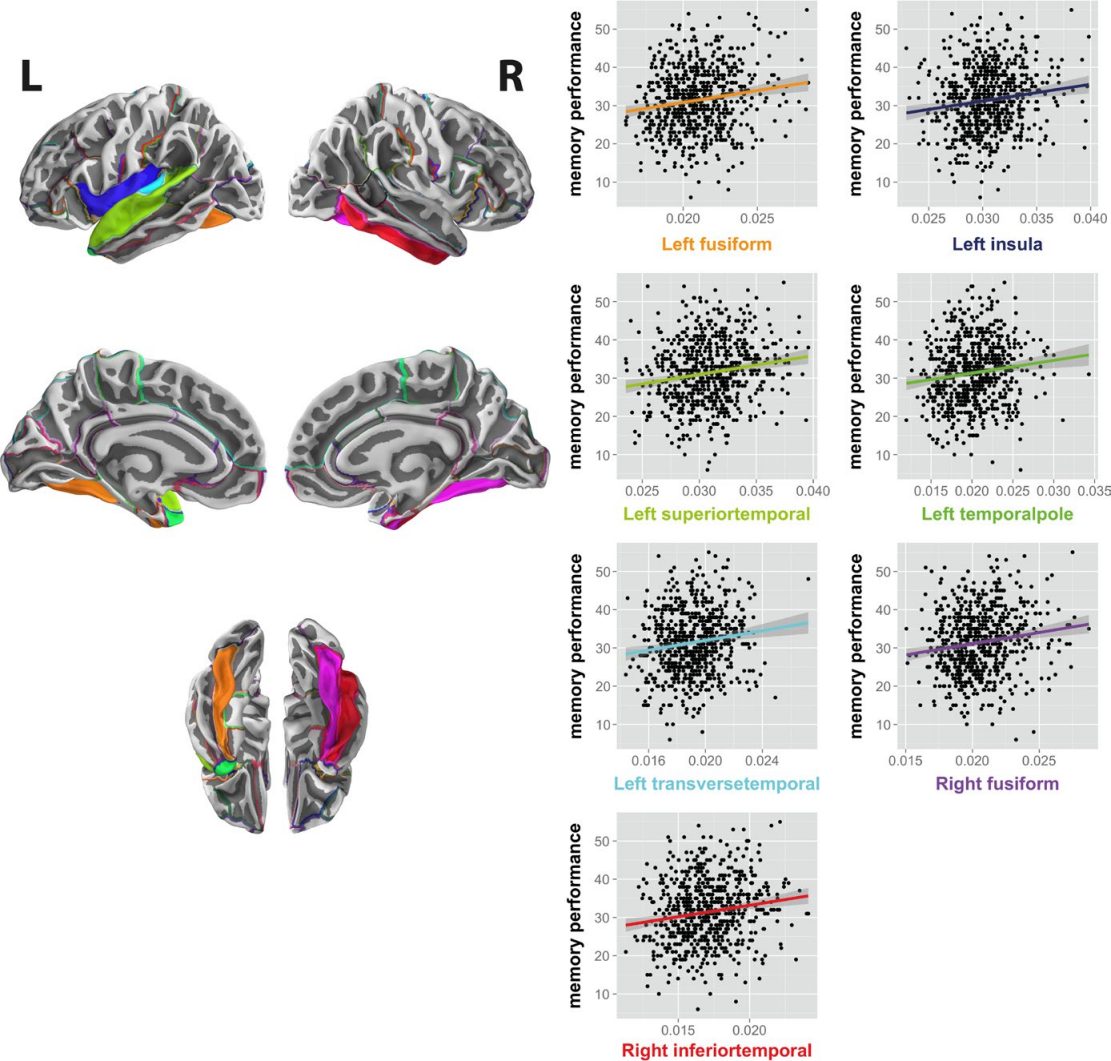
Recall‐relevant nodes. Nodes for which the degree (*i.e*. average connectivity of a node) was associated with picture recall performance at an FDR‐corrected level (*q *<* *.05). L: left; R: right

Next, we proceeded with the hierarchical approach (Figure 1b), and investigated nodal degree (*i.e*. the average connectivity of a node) with respect to an association with free recall performance. This revealed seven nodes whose degree was associated with recall performance at an FDR‐corrected (*q *<* *.05) level (Figure 3 and Table 1): the left fusiform gyrus, left superior temporal gyrus, left temporal pole, left transverse temporal cortex, the left insula, as well as the right fusiform gyrus and inferior temporal gyrus (see Table 2 for the complete distribution of correlation coefficients).

**Table 1:** List of nodes associated with picture recall performance, at an FDR‐corrected level (*q *<* *.05)

NODES	Association with picture recall		
	*r*	*p*	robust CI
Left fusiform	.115	.0030	0.04,0.19
Left superiortemporal	.149	1.12E‐04	0.08,0.22
Left temporalpole	.121	.0018	0.04,0.20
Left transversetemporal	.124	.0013	0.05,0.20
Left insula	.117	.0025	0.04,0.19
Right fusiform	.140	2.86E‐04	0.07,0.22
Right inferiortemporal	.143	2.15E‐04	0.07,0.22

The FDR‐corrected critical *p*‐value was *p *=* *.0032. *r*: Spearman correlation coefficient; *p*: nominal *p*‐value; *CI*: 95% percentile bootstrap confidence interval.

**Table 2:** Associations between picture recall performance and node degree or node gray matter volume

Node	Association with node degree		Association with gray matter volume	
	*r*	*p*	*r*	*p*
Left superiortemporal	.149	1.12E‐04	.008	8.44E‐01
Right inferiortemporal	.143	2.15E‐04	.011	7.75E‐01
Right fusiform	.140	2.86E‐04	.007	8.54E‐01
Left transversetemporal	.124	1.33E‐03	.027	4.88E‐01
Left temporalpole	.121	1.80E‐03	.033	4.02E‐01
Left insula	.117	2.45E‐03	.059	1.30E‐01
Left fusiform	.115	2.98E‐03	.007	8.52E‐01
Right temporalpole	.113	3.46E‐03	.016	6.85E‐01
Left Accumbens area	.103	7.88E‐03	.008	8.28E‐01
Right parahippocampal	.090	1.98E‐02	.067	8.25E‐02
Right insula	.088	2.30E‐02	.046	2.39E‐01
Right Hippocampus	.087	2.43E‐02	.030	4.38E‐01
Left isthmuscingulate	.083	3.15E‐02	.020	6.05E‐01
Right transversetemporal	.081	3.72E‐02	.069	7.59E‐02
Right Putamen	.080	3.96E‐02	.047	2.24E‐01
Right lateraloccipital	.078	4.56E‐02	.016	6.81E‐01
Right superiortemporal	.077	4.74E‐02	.001	9.83E‐01
Right precentral	.075	5.29E‐02	.016	6.87E‐01
Left supramarginal	.075	5.34E‐02	.014	7.25E‐01
Left bankssts	.073	6.07E‐02	.054	1.67E‐01
Left Thalamus Proper	.072	6.24E‐02	.032	4.11E‐01
Right inferiorparietal	.072	6.30E‐02	.019	6.34E‐01
Left lateralorbitofrontal	.071	6.82E‐02	.043	2.65E‐01
Right postcentral	.069	7.50E‐02	.042	2.85E‐01
Right Accumbens area	.066	8.95E‐02	.032	4.04E‐01
Left caudalanteriorcingulate	.066	9.17E‐02	.014	7.23E‐01
Right Amygdala	.065	9.23E‐02	.011	7.75E‐01
Left precuneus	.065	9.47E‐02	.040	3.09E‐01
Right bankssts	.064	9.75E‐02	.040	3.05E‐01
Right Caudate	.064	1.01E‐01	.029	4.55E‐01
Left Hippocampus	.063	1.07E‐01	.016	6.88E‐01
Left entorhinal	.062	1.08E‐01	.102	8.65E‐03
Right caudalmiddlefrontal	.061	1.15E‐01	.078	4.33E‐02
Left middletemporal	.060	1.23E‐01	.043	2.66E‐01
Left inferiorparietal	.059	1.27E‐01	.017	6.60E‐01
Right entorhinal	.057	1.43E‐01	.070	7.18E‐02
Left Pallidum	.053	1.72E‐01	.020	5.99E‐01
Left posteriorcingulate	.050	1.95E‐01	.012	7.55E‐01
Right rostralmiddlefrontal	.050	2.00E‐01	.013	7.42E‐01
Right posteriorcingulate	.049	2.10E‐01	.010	7.97E‐01
Left Putamen	.044	2.56E‐01	.037	3.44E‐01
Left lingual	.043	2.66E‐01	.052	1.78E‐01
Left parahippocampal	.039	3.16E‐01	.002	9.69E‐01
Right precuneus	.039	3.17E‐01	.043	2.65E‐01
Right superiorparietal	.038	3.28E‐01	.007	8.64E‐01
Right middletemporal	.038	3.30E‐01	.051	1.87E‐01
Right caudalanteriorcingulate	.037	3.40E‐01	.053	1.75E‐01
Right paracentral	.036	3.55E‐01	.041	2.95E‐01
Right isthmuscingulate	.033	3.98E‐01	.044	2.63E‐01
Left precentral	.033	4.00E‐01	.079	4.14E‐02
Right parsorbitalis	.032	4.15E‐01	.029	4.50E‐01
Left postcentral	.030	4.40E‐01	.025	5.17E‐01
Left parstriangularis	.030	4.44E‐01	.045	2.49E‐01
Left inferiortemporal	.028	4.68E‐01	.036	3.56E‐01
Right lateralorbitofrontal	.027	4.80E‐01	.036	3.58E‐01
Left paracentral	.026	5.03E‐01	.028	4.64E‐01
Left parsorbitalis	.025	5.25E‐01	.026	5.00E‐01
Right medialorbitofrontal	.024	5.44E‐01	.007	8.63E‐01
Left lateraloccipital	.023	5.47E‐01	.018	6.41E‐01
Left medialorbitofrontal	.022	5.77E‐01	.013	7.29E‐01
Right Thalamus Proper	.020	6.07E‐01	.049	2.11E‐01
Left Amygdala	.020	6.09E‐01	.062	1.09E‐01
Left Caudate	.019	6.26E‐01	.024	5.41E‐01
Left pericalcarine	.017	6.59E‐01	.066	8.93E‐02
Right supramarginal	.017	6.62E‐01	.041	2.96E‐01
Right cuneus	.016	6.82E‐01	.036	3.57E‐01
Left rostralanteriorcingulate	.015	7.05E‐01	.021	5.93E‐01
Left parsopercularis	.014	7.18E‐01	.008	8.39E‐01
Right lingual	.013	7.39E‐01	.069	7.36E‐02
Left caudalmiddlefrontal	.012	7.59E‐01	.020	6.01E‐01
Left rostralmiddlefrontal	.011	7.86E‐01	.015	6.99E‐01
Right parstriangularis	.010	7.99E‐01	.006	8.76E‐01
Right Pallidum	.009	8.07E‐01	.017	6.57E‐01
Left superiorfrontal	.009	8.14E‐01	.059	1.27E‐01
Right parsopercularis	.008	8.30E‐01	.032	4.06E‐01
Left superiorparietal	.008	8.44E‐01	.020	6.10E‐01
Left frontalpole	.007	8.55E‐01	.027	4.92E‐01
Right superiorfrontal	.007	8.65E‐01	.087	2.46E‐02
Right frontalpole	.006	8.85E‐01	.023	5.47E‐01
Left cuneus	.005	9.06E‐01	.094	1.50E‐02
Right pericalcarine	.001	9.81E‐01	.050	2.00E‐01
Right rostralanteriorcingulate	.000	9.99E‐01	.000	9.94E‐01

Nodes highlighted in bold are those for which the association between memory performance and node degree was significant at an FDR‐corrected level (*q *<* *.05); whereas nodes highlighted in italics were nominally associated. *r*: Spearman correlation coefficient; *p*: nominal *p*‐value.

Based on prior evidence of the influence of hippocampus‐related white‐matter connectivity on memory performance (Metzler‐Baddeley, Jones, Belaroussi, Aggleton, & O'Sullivan, 2011), and on a post‐hoc analysis on hippocampal degree, we found a significant association between memory performance and right hippocampus degree (*r *=* *.087; *p*_*nominal*_=.024). In total, the degree of 17 nodes were nominally associated with free recall, including contralateral regions to the FDR‐corrected nodes (temporal pole, insula, transverse temporal, superior temporal; see Table 2 and Supplementary Figure 1). The right hippocampus ranked at the 12th position.

### 3.3 Region‐to‐region level

**Figure 4:**
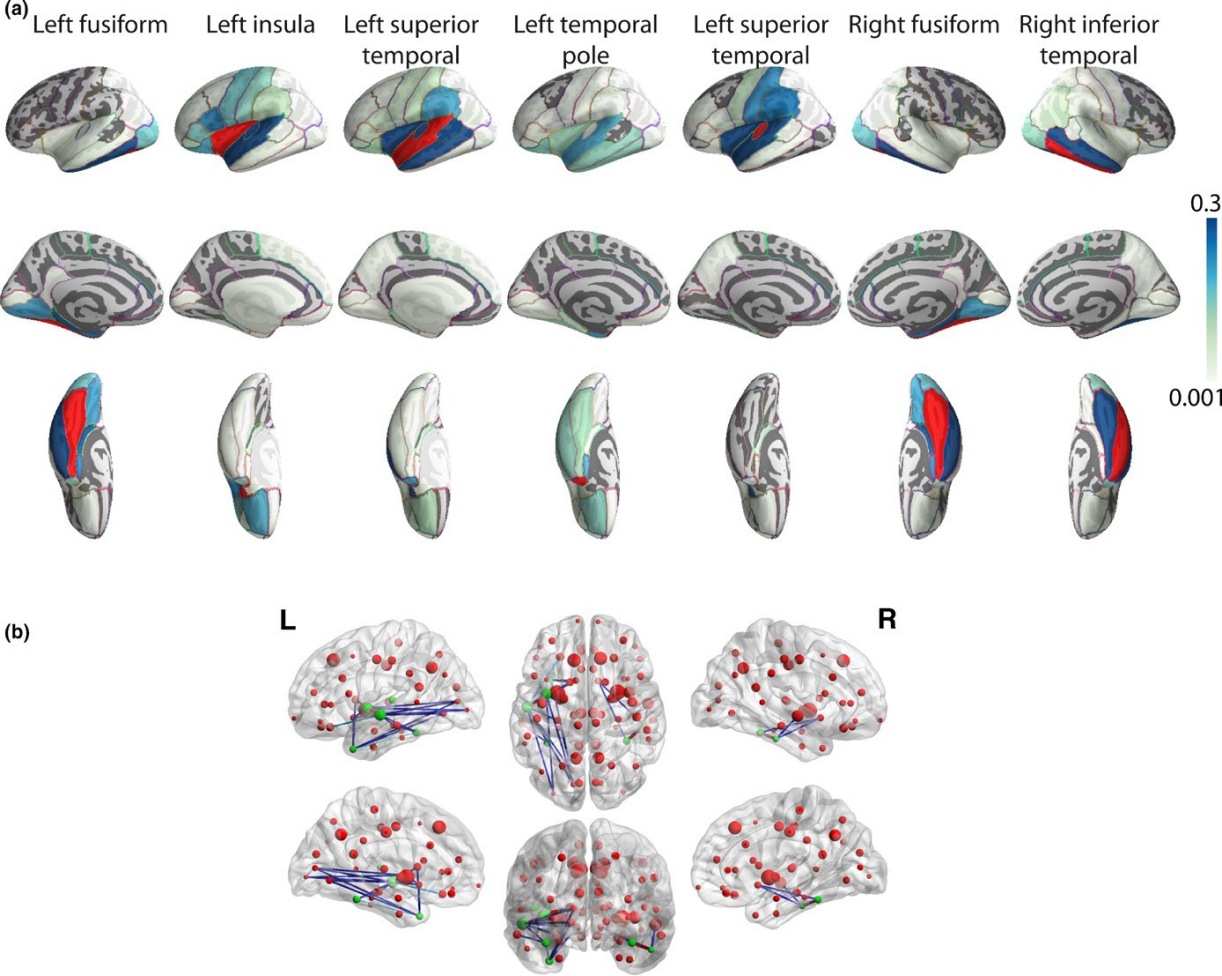
Recall‐relevant edges. (a) Whole‐brain connectivity pattern of the seven nodes associated with picture recall performance at an FDR‐corrected level (*q *<* *.05). The seed node is depicted in red. (b) Connections significantly associated with picture recall performance (FDR‐corrected level *q *<* *.05). The size of a node is proportional to its degree. Green nodes are those for which degree was significantly associated with picture recall performance (Figure 3). L: left; R: right

The final level in the hierarchical analysis consisted of investigating the node‐to‐node connectivity profile (*i.e*. the edges of the connectivity matrix) of the seven FDR‐corrected nodes. Out of the possible 551 edges that represent the connectivity profile of the FDR‐corrected nodes to all other nodes, 170 of the edges were considered after rejection of false‐positive connections (see Methods: *Structural brain network construction: population level, discarding spurious connections;* Figure 4a). Among these 170 edges, 22 connections were associated with free recall performance at an FDR‐corrected level (*q *<* *.05, Figure 4b, Table 3). Four of these 22 connections were between the seven FDR‐corrected nodes, which represent 40% of the existing connections between them, whereas the remaining 18 connections represented 11.2% of all other edges. A robust multiple regression model including individual values of these 22 connections accounted for 7.8% of the variance in picture recall performance.

**Table 3:** List of edges associated with picture recall performance, at an FDR‐corrected level (*q *<* *.05)

FDR edges		Association with picture recall		
FDR node	Connecting node	*r*	*p*	robust CI
Left fusiform	Left superiortemporal	.130	7.95E‐04	0.05,0.20
Left fusiform	Left temporalpole	.114	.0033	0.04,0.19
Left fusiform	Left middletemporal	.107	.0057	0.03,0.19
Left insula	Left pericalcarine	.133	5.76E‐04	0.06,0.21
Left insula	Left lateraloccipital	.121	.0018	0.04,0.20
Left insula	Left accumbens‐area	.120	.0020	0.04,0.20
Left superiortemporal	Left lingual	.189	8.87E‐07	0.11,0.26
Left superiortemporal	Left pericalcarine	.145	1.79E‐04	0.07,0.21
Left superiortemporal	Left accumbens‐area	.139	3.21E‐04	0.07,0.21
Left superiortemporal	Left insula	.132	6.30E‐04	0.06,0.21
Left superiortemporal	Left lateraloccipital	.130	8.09E‐04	0.05,0.20
Left superiortemporal	Left lateralorbitofrontal	.123	.0015	0.05,0.20
Left superiortemporal	Left hippocampus	.119	.0021	0.04,0.19
Left superiortemporal	Left caudate	.105	.0066	0.03,0.18
Left temporalpole	Left lingual	.145	1.70E‐04	0.06,0.22
Left temporalpole	Left accumbens‐area	.134	5.60E‐04	0.06,0.21
Left temporalpole	Left pericalcarine	.122	.0016	0.05,0.19
Left temporalpole	Left caudate	.112	.0039	0.04,0.19
Right fusiform	Right accumbens‐area	.116	.0027	0.04,0.19
Right fusiform	Right inferiortemporal	.115	.0030	0.04,0.19
Right inferiortemporal	Right superiortemporal	.114	.0032	0.04,0.18
Right inferiortemporal	Right accumbens‐area	.106	.0063	0.03,0.18

The FDR‐corrected critical *p*‐value was *p *=* *.0076. *r*: Spearman correlation coefficient; *p*: nominal *p*‐value; *CI*: 95% percentile bootstrap confidence interval.

### 3.4 Cost‐integrated metrics

As a further characterization of structural networks, we investigated several key topological metrics. Interindividual differences in memory performance were related to interindividual cost differences. Therefore, we investigated cost‐integrated topological values rather than standard weighted topological metrics (see Figure 1b) (Ginestet et al., 2011). No significant association was found between recall performance and cost‐integrated measures, nor did we find any significant valence‐by‐topological metric interaction (Table 4).

**Table 4:** Association between picture recall performance and cost‐integrated topological metrics

Global cost integrated values (over nodes)	Association with picture recall				
	Overall performance			Valence interaction	
	*r*	*p*	*robust CI*	*F*	*p*
Global efficiency	.013	.73	−0.061,0.086	0.39	.68
Clustering coefficient	−.025	.53	−0.093,0.054	0.39	.68
Characteristic path length (a)	.057	.14	−0.020,0.133	2.22	.11
Betweenness centrality	.013	.73	−0.064,0.090	0.76	.47

No metric survived correction for multiple comparison at an FDR of 5%. *r*: Spearman correlation coefficient; *p*: nominal *p*‐value; *CI*: 95% percentile bootstrap confidence interval.

a Computed on a sub‐sampled cost range for computational efficiency, due to the large number of random graphs to generate.

### 3.5 Network‐based statistics

The region‐to‐region level results were compared to a mass‐univariate approach working directly at the connection level, provided by the network‐based statistics toolbox. It identified one connected component associated with picture recall performance, at *p *=* *.0016 (FWE‐controlled). This component consisted of 23 nodes and 30 edges, and included left‐sided nodes representing mainly occipito‐temporal, insular, and temporo‐frontal connections. At the nodal level, there was an overlap of 50% between those 23 nodes and the 16 nodes forming the edges in the hierarchical approach, including five of the seven FDR‐corrected nodes (Table S1). At the connection level, 36.8% of the 30 NBS‐based connections were identical to those that the hierarchical approach detected (Figure S2 and Table S1). The NBS approach has been shown to be dependent on the initial cluster‐forming threshold. We therefore report the results of two additional analyses in the Tables S2 and S3, using a more stringent and a more lenient initial threshold (*T *=* *3 and *T *=* *2, respectively). A single FWE‐controlled component related to recall performance was identified in both cases (*p *=* *.0033 and *p *=* *.0001, respectively). The more liberal threshold component included 140 edges, among which all 22 of the hierarchical approach. The more stringent threshold component included nine edges, focusing on occipito‐ and fronto‐temporal connections. This independent approach supports our findings on the role that connections in occipito‐temporal regions play in explaining interindividual variability in memory performance.

### 3.6 Brain volumetry

Interindividual differences in memory recall have often been investigated in terms of brain volumetry (Van Petten, 2004). We therefore investigated whether or not individual grey matter volumes were associated with picture recall performance. We did so by means of a FreeSurfer‐based volumetric approach. We found neither a significant association for average volume (*r *=* *.05; *p *=* *.196), nor for regional volumes (FDR level *q *<* *.05; Table 2).

## 4 DISCUSSION

The present study revealed that variability of free recall performance was associated with variability in network density, a property of global connectivity that has been shown to be important not only in the characterization of structural networks (Gong et al., 2009), but also in functional networks related to memory (Watrous et al., 2013). Global structural connectivity has been shown to decrease with increasing age (Gong et al., 2009). This finding led to speculation that decreased global connectivity might represent an underlying factor for age‐related cognitive decline. By demonstrating that picture recall is positively correlated with global connectivity in young adults, the present results support the notion that decreased global structural connectivity might be involved in the age‐related decline in episodic memory performance. Moreover, we found evidence indicating that the association between structural connectivity and memory performance might be specific to the episodic task, as it did not apply to attention or working memory. As Schedlbauer et al. (Schedlbauer, Copara, Watrous, & Ekstrom, 2014) found an association between global functional network density and successful memory retrieval in a spatiotemporal task, these findings also point toward the functional relevance of network density in episodic memory. More generally, increased functional connectivity in a recollection‐specific network has also been related to recollection accuracy (King, de Chastelaine, Elward, Wang, & Rugg, 2015).

A post‐hoc analysis revealed that the degree of the right hippocampus, *i.e*. its average connectivity, was significantly associated with recall performance. Such a nominal association was observed for 20% of the nodes, reflecting the network density findings. Together with the whole‐brain hierarchical approach that identified its connection with the superior temporal cortex as relevant in explaining interindividual differences in recall performance, these findings confirm the role of the hippocampus as a key region for episodic memory (Milner & Penfield, 1955; Schacter & Tulving, 1994; Squire & Alvarez, 1995) in healthy young adults.

The whole‐brain hierarchical approach additionally allowed us to identify seven nodes, mainly located in the temporal lobe, whose degree was significantly associated with free recall performance. The fusiform gyrus, together with the inferior temporal cortex and the temporal pole, are part of the inferior longitudinal fasciculus, a fiber bundle that connects the occipital cortex with the anterior temporal lobe and the amygdala (Catani, Jones, Donato, & ffytche, 2003). Connections between these brain regions have been linked to episodic memory (Fuentemilla et al., 2009). Furthermore, age‐associated white matter injuries, as measured by white matter hyperintensities, were shown to be negatively associated with episodic memory performance in normal aging (Lockhart et al., 2012).

The network‐based statistics framework was employed as an independent confirmation approach. It identified one significant recall‐related component of left‐sided regions, including all five left‐sided nodes of our main analysis. Fourteen edges were identified as common between the two analyses. The fact that the NBS toolbox has distinct assumptions (mass‐univariate approach and connectedness of the components) offers a refined view on the main analyses. This approach highlights the role of connections of the superior temporal cortex and fusiform gyrus. Overall, these findings strengthen the importance of occipito‐temporal structural connections for episodic memory.

Not only do our results provide more specific information regarding connections previously related to memory performance, they also point to less investigated pathways, such as connections between the insula and the superior temporal cortex (Cerliani et al., 2012). Whereas the insula has a well‐known role in emotional processing (Craig, 2009), findings indicate that insular cortex infarction causes deficits in delayed verbal memory recall, which suggests that the insula is also a part of a functional network involved in episodic memory (Manes, Springer, Jorge, & Robinson, 1999). Furthermore, there is evidence that an abnormal insular functional network is associated with episodic memory decline in amnestic mild cognitive impairment (Xie et al., 2012) and the insular cortex is known to be important for memory acquisition and consolidation in rodents (Bermudez‐Rattoni, Ramirez‐Lugo, Gutierrez, & Miranda, 2004; Miranda & McGaugh, 2004). Our study also points to the importance of connections between the temporal pole and the nucleus accumbens, potentially mediated by the bed nucleus of the stria terminalis (Avery et al., 2014). Interestingly, studies in rodents have shown that the stria terminalis is involved in memory modulation (Roozendaal & McGaugh, 1996). So far, nothing has been known about the role of the stria terminalis in human memory.

Our results show that interindividual differences in memory performance were not explained by differences in grey matter volumes. Whereas grey matter volume differences have been linked to impaired memory performance when comparing cognitively impaired to healthy populations (Chételat et al., 2003), or young to old populations (Rajah, Kromas, Han, & Pruessner, 2010), less is known about the neuronal bases of interindividual variations in performance for young healthy subjects. Furthermore, in the latter case, the hippocampus has often been the main region of investigation (for a review see (Van Petten, 2004)). But results have been mixed, possibly due to low sample sizes or the heterogeneity of experimental protocols (Harrisberger et al., 2014; Van Petten, 2004). In contrast, our well‐powered study on whole‐brain structural correlates of picture recall provides evidence that interindividual differences in recall performance could not be explained by cortical or subcortical differences in grey matter volumes; rather, they can be explained by individual differences in the structural connections between grey matter structures.

Although the percentage of behavioral variance explained by structural connectivity measures might appear to be low, we think it represents a likely effect size, given the sample size of the present study (Button et al., 2013) and the dynamic nature of episodic memory processes. Structural connectivity represents the basis for the dynamic repertoire of functional interactions (Sporns, 2013) that could contribute to interindividual variability in behavior. Those interactions might further explain behavioral variance related to the different dynamic processes underlying episodic memory (encoding, consolidation, recall). Other factors that were not assessed in this study, such as IQ or education level, might also contribute to differences in memory performance. Another limitation is that a single measure of picture recall was employed. These points underscore the fact that replication of the present results with different episodic memory tasks and populations would be of particular interest.

Additional methodological limitations also have to be mentioned. The DWI sequence used in this study allowed us to scan a large cohort in an acceptable amount of time on a clinical scanner. Recent developments in terms of multi‐band imaging (Feinberg & Setsompop, 2013) could be beneficial to increase the angular resolution for such large‐scale studies, without increasing scanning time. Regarding the reconstruction of individual connectomes, we opted to estimate the voxel fiber orientations by using a model‐based approach (Behrens et al., 2007). Non‐parametric alternatives such as spherical deconvolution might refine the estimation in complex sub‐voxel fiber configurations (Lenglet et al., 2009). Tractography choices, ranging from node definition (de Reus & van den Heuvel, 2013) to tracking algorithms (Bastiani, Shah, Goebel, & Roebroeck, 2012; Girard, Whittingstall, Deriche, & Descoteaux, 2014) are also known to have an impact on connectome measures. Considering the lack of findings for cost‐corrected graph metrics, it is possible that the cost integration, combined with the hierarchical approach, masks isolated effects. We nevertheless consider this approach important regarding the problematic of correctly disentangling the effects of cost from those of topology in the connectome.

In conclusion, we report that interindividual differences in picture free recall performance are related to interindividual differences in global structural connectivity and connectivity between specific brain regions. Structural connections between the occipital and temporal lobes are of known functional relevance for memory processes, while connections between the temporal pole and the nucleus accumbens represent novel findings. The identification of connectome‐based neural correlates of interindividual differences in memory performance demonstrates the usefulness of this novel approach in characterizing complex cognitive traits. Such correlates could represent an interesting starting point for human genetic studies, and prove useful as endophenotypes of psychiatric disorders.

## CONFLICT OF INTEREST

The authors declare no competing financial interests.
