# Supplementary figures and images for: Picture free recall performance linked to the brain's structural connectome
Source: Brain Behav. 2017 May 23;7(7):e00721. doi: 10.1002/brb3.721 (PMC5516597; doi:10.1002/brb3.721)

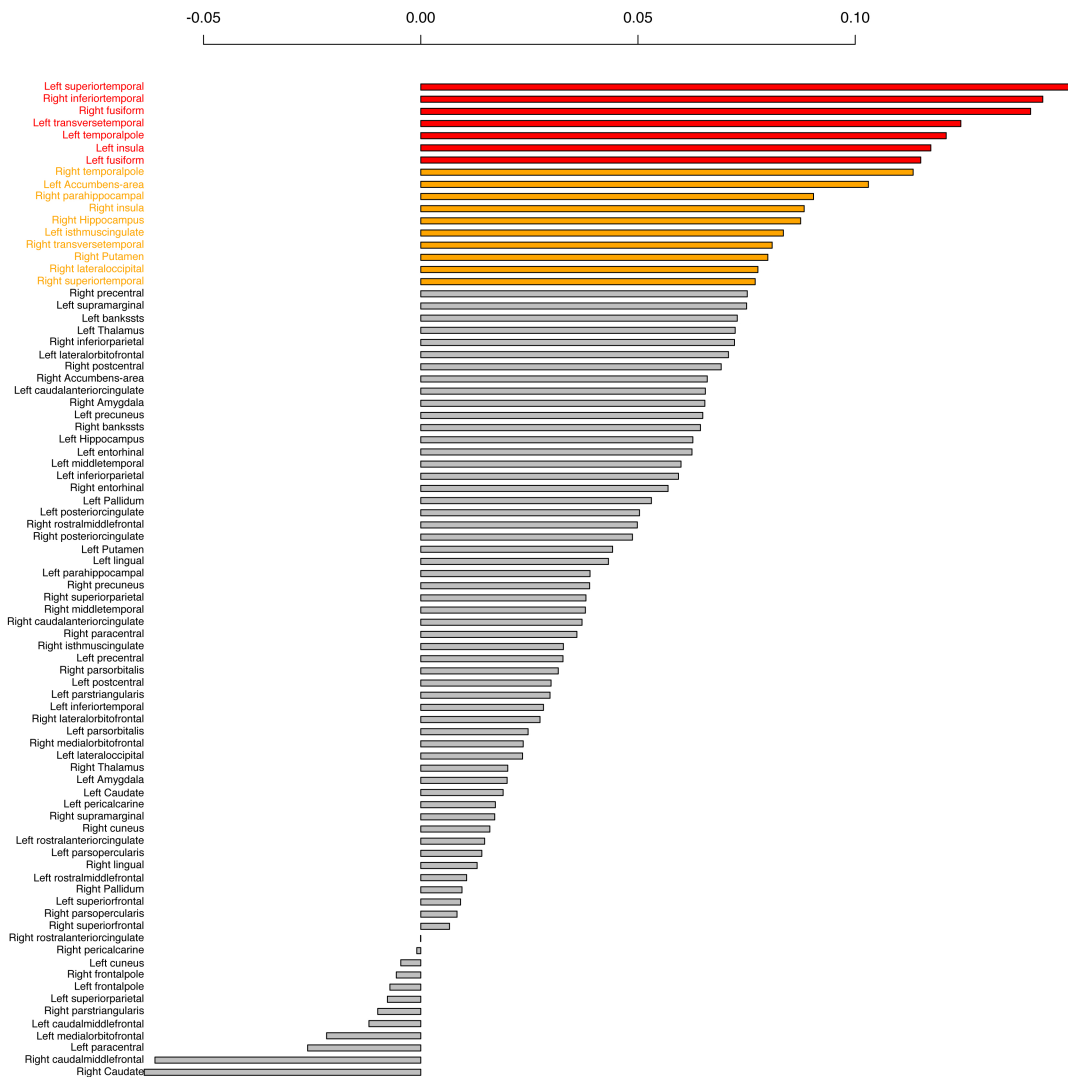

Supplement: Supplementary file 1 [file BRB3-7-e00721-s001.pdf]

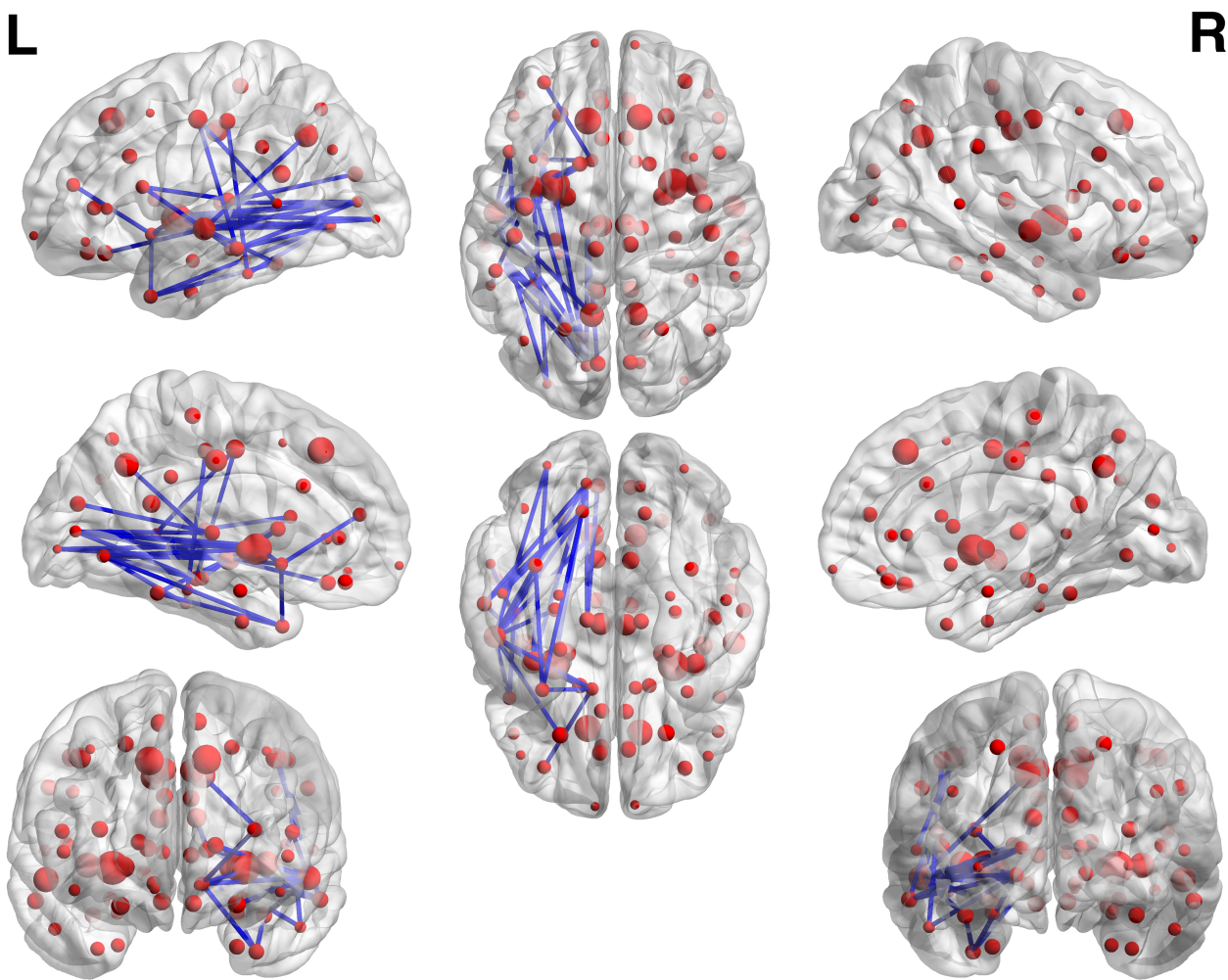

Supplement: Supplementary file 2 [file BRB3-7-e00721-s002.pdf]
